# Gut Microbiota Alterations in Heart Failure Patients: Insights from a Systematic Review

**DOI:** 10.3390/jcm14228110

**Published:** 2025-11-16

**Authors:** Néstor Báez-Ferrer, Alejandro Lemus-Martín, María Beatriz Castro-Hernández, Pablo Avanzas, Susana Martínez-González, María Lecuona-Fernández, Alberto Domínguez-Rodríguez

**Affiliations:** 1 Cardiology Department, Hospital Universitario de Canarias, 38320 Tenerife, Spainalexlemusmartin@gmail.com (A.L.-M.); 2 Microbiology Department, Hospital Universitario de Canarias, 38320 Tenerife, Spain; mbcasher@gobiernodecanarias.org (M.B.C.-H.); mlecfer@gobiernodecanarias.org (M.L.-F.); 3 Heart Area, Hospital Universitario Central de Asturias, Instituto de Investigación Sanitaria del Principado de Asturias, 33011 Oviedo, Spain; avanzas@gmail.com; 4 Medicine Department, University of Oviedo, 33003 Oviedo, Spain; 5 Centro de Investigación Biomédica en Red de Enfermedades Cardiovasculares (CIBERCV), 28029 Madrid, Spain; 6 Endocrinology, Hospital Universitario Nuestra Señora de la Candelaria, 38230 Tenerife, Spain; smargonz@gobiernodecanarias.org; 7 Department of Internal Medicine, University of La Laguna, 38200 Tenerife, Spain

**Keywords:** gut microbiota, heart failure, trimethylamine N-oxide, lipopolysaccharide, indoxyl sulfate, short-chain fatty acids

## Abstract

**Background/Objectives:** Heart failure (HF) is associated with chronic systemic inflammation, resulting in increased mortality. The intestinal microbiota can modulate systemic inflammation, and changes in the microbiota have been observed in patients with HF. **Methods:** A systematic search was performed in PubMed/MEDLINE up until July 2025 for studies comparing the intestinal microbiota between patients with HF and healthy controls (HCs). The PRISMA (Preferred Reporting Items for Systemic reviews and Meta-Analyses) criteria were used. The risk of bias was evaluated with the Newcastle–Ottawa scale for cross-sectional studies. **Results:** Fourteen studies with 1167 participants (550 patients with HF and 617 HC) were included. The patients with HF had less alpha and beta diversity compared with HC. In turn, the patients with HF presented an increase in proinflammatory bacteria belonging to the genera *Streptococcus* and *Escherichia-Shigella*, and a decrease in bacteria with anti-inflammatory effects, pertaining to the genera *Faecalibacterium*, *Blautia* and *Lachnospira*. **Conclusions:** Patients with HF present an altered intestinal microbiota, favoring the growth of bacteria that increase systemic inflammation through their metabolic activity. Modulation of the intestinal microbiota through different approaches is seen as a new therapeutic target in HF.

## 1. Introduction

Heart failure (HF) is a complex syndrome defined by a number of signs and symptoms resulting from structural or functional alterations of the heart, with an inability to maintain an adequate cardiac output and/or an increase in ventricular filling pressure [1]. Heart failure is one of the leading causes of morbidity and mortality, currently affecting 1–3% of the world’s population, and its prevalence is increasing due to progressive aging of the population, advances in treatment, and increased survival among patients with different cardiovascular disorders [2]. In turn, HF is associated with high healthcare costs, as it is one of the leading causes of hospital admission or close outpatient monitoring, which requires numerous diagnostic studies and the use of extensive therapeutic resources [3,4].

In recent years, there has been a growing interest in the study of the intestinal microbiota as part of the pathophysiology of inflammation found in different chronic diseases [5]. There are 10^14^ bacteria in the human intestine—ten times more than the number of cells in the body. Up to 1150 different bacterial species are known to potentially form part of the human intestinal microbiota, and each individual presents about 160 species [6]. In the general population, the predominant bacterial phyla that represent 90% of the intestinal flora are Bacillota, previously known as Firmicutes (e.g., *Clostridium*, *Faecalibacterium*, *Lactobacillus*, *Enterococcus*, *Staphylococcus*, *Streptococcus*), and Bacteroidota, previously known as Bacteroidetes (e.g., *Bacteroides*, *Prevotella*, *Parabacteroides*, *Alistipes*), while the remaining 10% are mainly represented by Actinomycetota, Pseudomonadota, Verrucomicrobiota, and Fusobacteriota [7,8,9]. The variability between individuals is due to a range of factors, including particularly the diet, environmental factors, age, genetics, and ethnicity [10,11]. Another modifier of the microbiota is the use of common drugs such as proton pump inhibitors or antibiotics [12].

The intestinal microbiota has key functions in the body. It participates in digestion, synthesizes certain vitamins and hormones, modulates the immune response, protects against external pathogens, and maintains the intestinal barrier. In addition, the microbiota produces many metabolites with effects at systemic level [13]. Dysbiosis is characterized by a modification or imbalance of the intestinal microbiota due to quantitative or qualitative alterations in its composition, and which affect its function [5]. Differences have been observed in the richness, composition, and distribution of the intestinal microbiota and its derived metabolites in patients with HF, leading to the term “gut–heart axis” introduced over a decade ago [14]. These observed differences in the intestinal microbiota composition are due to a lower alpha and beta diversity in patients with heart failure (HF) compared to healthy controls, suggesting lower bacterial richness and community variation [13]. The main taxonomic changes are based on a decrease in the phyla Bacillota and Bacteroidia, as well as an increase in the phyla Pseudomonadota and Actinobacteria [13]. These compositional changes lead to metabolic alterations with an altered production of gut-derived metabolites such as short-chain fatty acids (SCFAs), trimethylamine N-oxide (TMAO), and lipopolysaccharides (LPSs) [12]

The present systematic review was carried out to identify and summarize the available scientific evidence on the intestinal microbiota profile of patients with HF and its differences with respect to the healthy population.

## 2. Methods

This systematic review was conducted following the Preferred Reporting Items for Systematic Reviews and Meta-Analyses (PRISMA) 2020 guidelines. The PRISMA checklist was used to guide the reporting process and is available in the Supplementary Materials. This review was not registered [15]. A search was carried out of the PubMed/MEDLINE database up until July 2025 for relevant studies on the interaction of the intestinal microbiota with HF. The search strategy made use of MeSH terms and keywords combined using Boolean operators, based on the following equation: (“Heart Failure” OR “heart failure with reduced ejection fraction” OR “heart failure with preserved ejection fraction”) AND (“Gut microbiota” OR “intestinal microbiota” OR “gut dysbiosis” OR “microbiome” OR “gut flora”).

The review included all original studies on the intestinal microbiota using gene sequencing methods in patients with HF, published in English between July 2015 and July 2025. Inclusion criteria: This study included observational studies or clinical trials that provided clinical data and detailed taxonomic information, including genetic sequencing data, to enable a comprehensive analysis of gut microbiota composition and function. Only studies reporting strict and clearly specified numerical data, along with relevant clinical characteristics of patients with heart failure, were considered. Exclusion criteria: Studies were excluded if they did not analyze clinical data from patients or did not provide specific taxonomic information. In addition, articles with the following formats were excluded: letters to the editor, editorials, short communications, animal studies, narrative reviews, systematic reviews, meta-analyses, and studies involving interventions to modulate the microbiota.

The risk of bias (RoB) of each of the studies was evaluated independently by three of the authors, using the Newcastle–Ottawa scale for cross-sectional studies [16]. The total score classified the studies as presenting high RoB (0–3 points), moderate RoB (4–6 points) or low RoB (7–9 points).

The characteristics of the intestinal flora of the patients with HF were analyzed, and alpha and beta diversity and the differences in relative abundance of the microbiota were compared against a group of healthy controls (HCs) or against the patients with HF if these were followed-up on over time. Alpha diversity analyzes the richness or abundance of species in a sample, as well as their evenness or distribution. Beta diversity in turn assesses the differences in composition of the microbiota between two or more groups. Lastly, relative abundance refers to the percentage represented by each of the different taxa within the total microbiota.

A homogeneous statistical analysis could not be performed due to the heterogeneity of the studies. This study was conducted following the ethical principles of the Declaration of Helsinki on medical research in humans [17], and was approved by the Research Ethics Committee of Complejo Hospitalario Universitario de Canarias (Spain), under code CHUC_2024_76 (MICRO-CHF).

## 3. Results

### 3.1. Characteristics of the Participants and Studies Included in the Review

**Figure 1:**
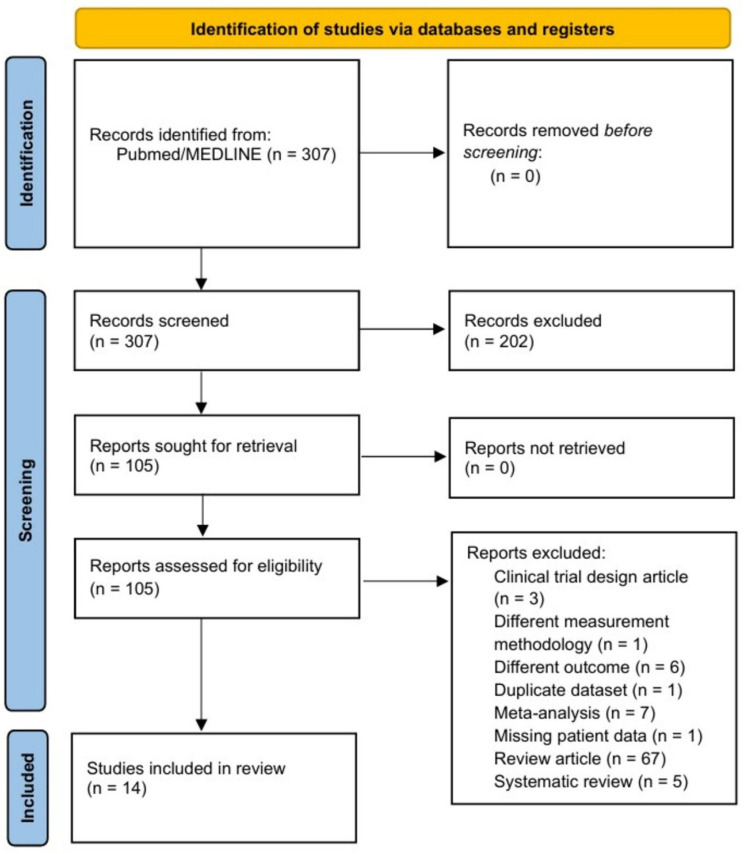
Flow chart of the literature search for studies on the intestinal microbiota in patients with heart failure.

The initial search identified 307 articles. Of these, 202 were excluded after reading the title and abstract. Following full-text reading and applying the exclusion criteria, 6 observational studies that were not focused on the objective of our review were discarded, along with 3 clinical trial protocols, 67 narrative reviews, 5 systematic reviews, and 7 meta-analyses. Finally, we excluded the study published by Pasini et al. (2016) [18] as it did not use gene sequencing; the article published by Kummen et al. (2018) [19] due to insufficient data on the included subjects; and the article published by Hayashi et al. (2021) [20], which involved the same database as that of a previous study from 2019, with a different objective. Thus, a total of 14 articles were finally included in this systematic review (Figure 1).

The total number of participants was 1167 (550 patients with HF and 617 controls). Table 1 shows the characteristics of the patients according to the different studies included. All the included articles were observational studies. Six of the studies (43%) were carried out in China [21,22,23,24,25,26], three (21%) in Japan [27,28,29], two (14%) in Australia [30,31], and three (21%) in Europe: one in Germany [32], another in Norway [33], and another in Spain [34]. Eight of the studies (57%) selected hospitalized patients for the HF group, while four (29%) selected outpatients. One study included both hospitalized patients and outpatients, while another failed to specify the origin of the patients.

There were 11 cross-sectional studies (78%) that analyzed the intestinal microbiota in patients with HF and controls at the time of inclusion [21,22,23,24,25,26,27,28,30,32,33]. Three studies (21%) analyzed the intestinal microbiota as paired data. Hayashi et al. (2019) [29] studied the intestinal microbiota as paired data during admission, both initially (decompensated phase) and in the compensated phase, establishing comparisons between them and against the control group. Ahmad et al. (2023) [31] in turn analyzed the intestinal microbiota of patients hospitalized with acute HF and of outpatients with chronic HF, establishing comparisons both jointly and separately against the control group at the start and after 6 months of follow-up. The study by Modrego et al. (2023) [34] lacked a control group and compared the intestinal flora of patients admitted due to onset of HF versus the intestinal bacterial growth observed after 12 months of follow-up.

Based on the left ventricular ejection fraction (LVEF) [21,28,31,32,33], five studies (36%) focused on patients with a reduced LVEF [21,28,31,32,33], and two (14%) on patients with a preserved LVEF [23,30], while seven (50%) did not take LVEF into account [22,24,25,26,27,29,34].

### 3.2. Methodology of the Studies Included in the Review

Identification of the bacterial species of the intestinal microbiota was made based on genetic sequencing methods using stool samples from the participants. Twelve studies (86%) employed 16S rRNA sequencing, amplifying different regions such as V1-V2, V3-V4, or V4-V5, depending on the study. With regard to the other two articles, Cui et al. (2018) [21] used metagenomic sequencing, while Wang et al. (2021) [22] used 16S rDNA sequencing to amplify the V3-V4 region.

### 3.3. Quality Assessment

The methodological quality of the studies included in the review ranged from moderate to low RoB. Six studies were classified as presenting moderate RoB, and eight as presenting low RoB. The main quality difference between the studies was attributable to the degree of adjustment for confounding factors in the inter-group comparisons, reflecting greater solidness of the low RoB studies in identifying robust associations (Table 1).

### 3.4. Alpha and Beta Diversity in HF Versus the Control Group

The analysis of alpha diversity was based on different statistical methods such as the observed Operational Taxonomic Units and the Phylogenetic Diversity Whole Tree, described by Shannon, Simpson, and Chao1. All but one of the studies conducted an analysis of alpha diversity. Five studies (36%) analyzed both richness and evenness; four (29%) analyzed global alpha diversity and specifically richness; two (14%) evaluated global alpha diversity with no specific analysis of richness or evenness; one (7%) reported global alpha diversity and specified evenness; and another study (7%) only analyzed richness (Table 2). Significant differences in alpha diversity were observed between the patients with HF and preserved LVEF and the controls, at the expense of less richness in the former group—though no changes in evenness were demonstrated. Regarding the patients with lowered LVEF, significant differences versus the controls were observed in three of the four studies that analyzed them: two due to a decrease in global alpha diversity and one due to less richness. The remaining study found no significant differences. Among the seven studies in which LVEF was not specified, two found no significant differences between the groups, and four recorded less alpha diversity in the HF group. The study by Modrego et al. (2023) [34] recorded no significant differences in the alpha diversity of the intestinal microbiota between onset and follow-up at 12 months.

Beta diversity was analyzed based on Bray–Curtis dissimilarity or weighted and unweighted UniFrac, and was graphically displayed by principal coordinate analysis or non-metric multidimensional scaling. Two of the studies did not evaluate beta diversity. Statistically significant differences in intestinal microbiota were demonstrated in the 11 studies that compared beta diversity between patients with HF and controls. No significant differences were found in terms of beta diversity between onset and follow-up at 12 months in the study published by Modrego et al. (2023) [34]. The results are shown in Table 2.

### 3.5. Differences in Relative Abundance of the Intestinal Microbiota in HF Versus the Control Group

**Figure 2:**
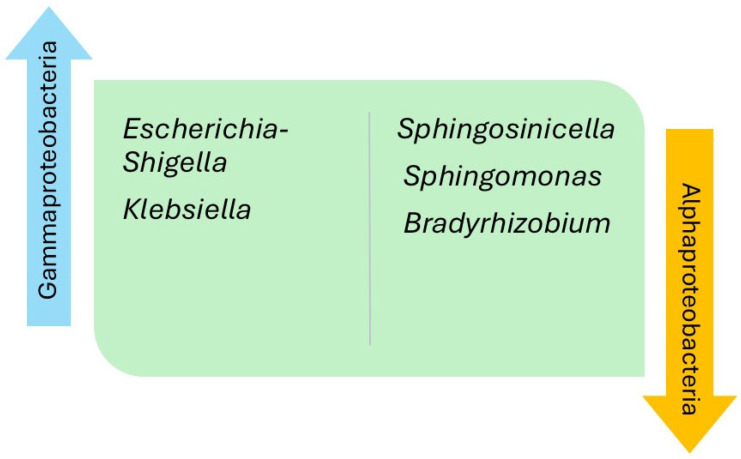
Differences in genera within the classes Bacilli and Clostridia, belonging to the phylum Bacillota, in patients with heart failure compared with healthy controls.

**Figure 3:**
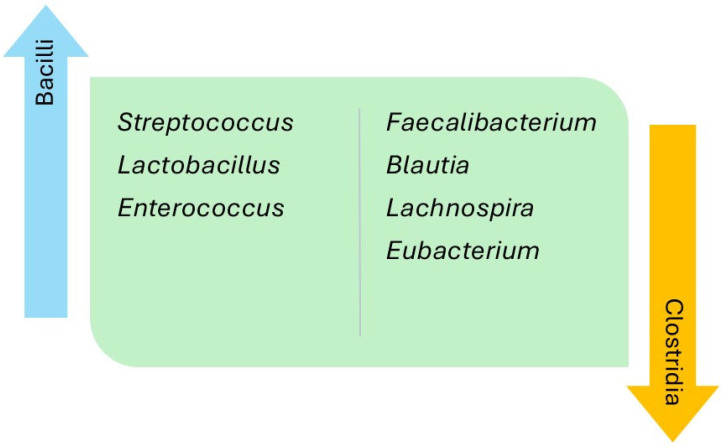
Differences in the genera of the classes Gammaproteobacteria and Alphaproteobacteria, belonging to the phylum Pseudomonadota, in patients with heart failure compared with healthy controls.

The relative abundance of the intestinal microbiota was analyzed and compared at different taxonomical levels (Table 3). Regarding the thirteen studies that established comparisons versus the controls, 29% reported a decrease in the phylum Bacillota in the HF group, while the phyla Actinomycetota, Pseudomonadota, and Synergistota were found to be increased in three (21%), two (14%), and one (7%) of the studies, respectively. The class Clostridia, belonging to the phylum Bacillota, was seen to be decreased in the HF group in two studies (14%), and the class Alphaproteobacteria, within the phylum Pseudomonadota, was seen to be increased in one of them (7%). Of note, in relation to the class Clostridia, was a decrease in the relative abundance of the families Lachnospiraceae and Ruminococcaceae in the HF group versus the controls in 30% and 21% of the studies, respectively. However, one of the studies reported an increase in abundance of the family Ruminococcaceae in patients with HF versus the controls. The main findings regarding genus differences corresponded to the classes Bacilli and Clostridia of the phylum Bacillota, and the class Gammaproteobacteria pertaining to the phylum Pseudomonadota. The phylum Bacillota showed a greater relative abundance in the HF group versus the controls corresponding to various bacteria of the class Bacilli, at the expense of the genera *Streptococcus* (46%), *Lactobacillus* (38%), and *Enterococcus* (15%), as well as a lesser relative abundance of the class Clostridia, at the expense of the genera *Faecalibacterium* (38%), *Blautia* (23%), *Lachnospira* (15%), and *Eubacterium* (15%) (Figure 2). The phylum Pseudomonadota showed a greater relative abundance of bacteria pertaining to the class Gammaproteobacteria among the patients with HF versus the controls, at the expense of the genera *Escherichia*-*Shigella* (38%) and *Klebsiella* (23%), as well as a lesser relative abundance of bacteria belonging to the class Alphaproteobacteria, at the expense of the genera *Sphingosinicella*, *Sphingomonas*, and *Bradyrhizobium* (Figure 3). The genera *Ruminococcus* (phylum Bacillota) and *Bifidobacterium* (phylum Actinomycetota) showed variable results between the studies. Lastly, only two studies analyzed the different species between patients with HF versus the controls. The HF group showed the highest relative abundance of *Ruminococcus gnavus*, *Streptococcus* sp., and *Veillonella* sp., and a decrease in *Eubacterium rectale*, *Dorea longicatena*, *Faecalibacterium prausnitzii*, *Oscillibacter* sp., and *Sutterella wadsworthensis*. In Table 4, we summarize the findings regarding the percentages of taxonomic levels reported in the studies.

The studies that compared the microbiota as paired data during different phases of the disease recorded some differences in relative abundance. Ahmad et al. (2023) [31] observed an increase in the genus *Lacticaseibacillus* (class Bacilli, phylum Bacillota) in patients with chronic HF versus those with acute HF at the time of inclusion in the study, and found the genera *Prevotella 7*, *Megamonas*, and *Libanicoccus* and bacteria of the family Barnesiellaceae to be the organisms that most distinguished the microbiota of the chronic HF group from that of the acute HF group after 6 months of follow-up. At 12 months of follow-up after the onset of HF, Modrego et al. (2023) [34] detected a decrease in the genera *Pectobacterium*, *Sphingosinicella*, *Sphingomonas*, and *Bradyrhizobium* corresponding to phylum Pseudomonadota, as well as in the family RF-39 of the phylum Bacillota.

Additionally, we have described possible factors that may contribute to the modulation of the gut microbiota in patients with heart failure (Table 5). Most studies excluded participants who had used probiotics or antibiotics in the preceding months. Medications that could affect the microbiota, such as proton pump inhibitors and diuretics, were reported in only a few studies, preventing any meaningful conclusions. Only five studies specified the diet of the participants; however, dietary patterns differed across these studies, which precluded definitive conclusions. Table 6 shows the main differences in metabolites derived from gut microbiota.

## 4. Discussion

The present systematic review describes for the first time the results obtained by the different studies published in the literature, grouped by taxonomic levels. The intestinal microbiota of patients with HF and the healthy controls showed less alpha and beta diversity, independently of LVEF, and a different relative abundance between the individuals with HF versus the controls—thus supporting the existence of a gut–heart axis [14]. The main differences in intestinal microbiota between patients with HF and the healthy controls were related to the phyla Bacillota and Pseudomonadota. Within the phylum Bacillota, a decrease was observed in most of the genera of the class Clostridia, particularly *Faecalibacterium*, as well as an increase in the class Bacilli, particularly of the genera *Streptococcus* and *Lactobacillus*. With regard to the phylum Pseudomonadota, the patients with HF mainly showed an increase in the genera *Escherichia-Shigella* and *Klebsiella*, pertaining to the class Gammaproteobacteria, with respect to the controls.

In this review, 75% of the studies comparing alpha diversity in the HF group versus the controls observed significant differences, while the three studies that failed to identify differences were those with the smallest control sample sizes. These differences reflect less alpha diversity in the HF group due to both lower richness and less evenness. The present review demonstrates a clear difference in the composition of the intestinal flora in patients with HF versus the controls, independently of LVEF, as reflected by the differences in beta diversity found in all the studies where it was analyzed. Modrego et al. (2023) [34] found no significant differences in alpha and beta diversity between admission and 12 months of follow-up in the patients with HF. However, this study involved a small cohort of only 18 patients, 2 of which moreover died before one year; as a result, their stool samples were not included in the analysis.

### 4.1. Inflammation and Intestinal Microbiota in Heart Failure

Individuals with HF present chronic activation of inflammatory processes, with an increase in proinflammatory cytokines such as tumor necrosis factor-alpha (TNF-α), interleukin-1, or interleukin-6 (IL-6) [35]. Increased inflammation has been associated with greater mortality in patients with HF [36]. However, relevant aspects of the pathophysiology of inflammation in HF remain to be clarified, and some authors have identified dysbiosis as an intervening factor in systemic inflammation [37,38,39].

Intestinal ischemia or splanchnic congestion may result from both a decrease in cardiac output and systemic venous congestion, defined by an increase in central venous pressure. This in turn alters the blood–intestinal barrier, increasing its permeability and leading to greater bacterial translocation and an increase in circulating endotoxins such as lipopolysaccharides (LPSs)—activating systemic inflammatory mechanisms [40,41]. The intestinal villi are very sensitive to the described hemodynamic changes, which favor the anaerobic route and result in intracellular acidosis. The latter in turn activates sodium-hydrogen exchanger 3 (NHE-3). NHE3 facilitates greater sodium absorption, which can perpetuate volume overload and worsen systemic congestion. It moreover leads to the release of hydrogen ions (H+) into the intestinal lumen, modifying the habitat of the intestinal microbiota and producing changes in its composition and metabolism [42]. Recently, attention has been drawn to the role of the Paneth cells, which also suffer the changes produced by the aforementioned congestion and hypoperfusion, and which are implicated in the maintenance and regulation of the intestinal barrier through recognition of the intestinal microbiota by toll-like receptors and the secretion of antimicrobial peptides [43].

This change in the intestinal environment alters the composition of the microbiota, favoring the proliferation of harmful strains and reducing the presence of beneficial species. These bacteria in turn produce metabolites that reach the systemic circulation—some exerting a protective role while others may perpetuate HF [44]. These metabolites include TMAO, SCFA [13], LPS, and indoxyl sulfate (IS) [45].

### 4.2. Metabolites and Inflammation Derived from the Different Bacterial Genera in Heart Failure

Regarding the bacterial populations, almost half of the studies reported an increase in the genus *Streptococcus* in the HF group versus the controls. This is a relevant finding, since this genus contains peptidoglycan and lipoteichoic acid (LTA) in its structure, both of which can generate a systemic inflammatory response on being recognized by TLR2 and stimulating the nuclear factor kappa-B (NF-κB) pathway [46]. Moreover, the bacteria of this genus have been shown to be able to cross the intestinal barrier through different mechanisms that affect the intercellular junctions of the digestive tract [47,48].

Other genera found to be increased in patients with HF in this review and which are of relevance due to their proinflammatory potential are *Escherichia-Shigella*, *Klebsiella*, *Prevotella*, and *Acinetobacter*. All of them are Gram-negative bacteria containing LPS (also known as endotoxin) in their outer membrane. The structure of LPS is based on the binding of an oligosaccharide with lipid A. It exerts proinflammatory activity through different pathways such as NF-kB and the innate immune response, associated with high levels of proinflammatory cytokines TNF-α and IL-6. Furthermore, lipid A is able to bind to TLR4, which through different proinflammatory pathways can cause myocardiocyte apoptosis and generate myocardial fibrosis [49,50]. Endotoxinemia has been associated with greater systemic congestion in patients with HF [51]. In addition, patients with acute HF have shown a difference in LPS concentration between the hepatic vein and the left ventricle, suggesting a splanchnic origin of the endotoxin [52].

Using precursors found in the diet, such as choline, L-carnitine, and betaine, 80% of the bacteria conforming the intestinal microbiota are able to produce trimethylamine (TMA), which is then transformed into TMAO in the liver [53]. Bacteria of the family Enterobacteriaceae have been identified as the main producers of TMA, including those belonging to the genera *Escherichia* and *Klebsiella* [54], both of which are increased in patients with HF, as seen in this review. Hayashi et al. (2019) [29] reported a positive correlation between the levels of TMAO and the concentrations of *Escherichia-Shigella*. TMAO is recognized as a promoter of inflammatory responses through different pathways [55]. On the other hand, through the activation in the myocardiocytes of growth factor β1 and the Smad proteins, which increase transcription of the genes related to atrial natriuretic peptide and the heavy chain of β-myosin, it is capable of promoting left ventricular hypertrophy and myocardial fibrosis [56]. Tang et al. (2014) [57] demonstrated increased TMAO levels in patients with HF compared with the healthy controls, as well as a positive correlation between the levels of TMAO and the concentration of type B natriuretic peptide. After 5 years of follow-up, they found increased TMAO levels in patients with HF to be associated with a 3.4-fold higher mortality rate. A meta-analysis involving 6879 patients with HF and a follow-up period of 5 years corroborated these data, with an increase in major cardiovascular events (hazard ratio [HR] 1.68; 95% CI, 1.44–1.96) and mortality (HR 1.67; 95% CI, 1.17–2.38) in those patients with elevated TMAO [58]. Another recent meta-analysis involving 3300 individuals has reported a strong inversely proportional association between the TMAO levels and LVEF [59].

Another molecule associated with the genus *Escherichia* is IS, which is derived from tryptophan, and has been shown to be increased in patients with HF [60]. The same research group added prognostic value to this metabolite, recording an increased incidence of cardiovascular events (death due to cardiovascular causes or hospital admission) with rising plasma IS levels in a cohort of patients with HF at 5 years (HR 1.84; 95% CI, 1.28–2.51) [61].

On the other hand, the present review has found that patients with HF present a lower relative abundance of the genera *Faecalibacterium*, *Blautia*, *Megamonas*, *Lachnospira*, and *Eubacterium*, and of the species *Eubacterium rectale*, *Dorea longicatena*, *Faecalibacterium prausnitzii*, and *Oscillibacter* sp., which are bacteria that produce SCFAs as metabolites derived from the bacterial fermentation of dietary fiber in the digestive tract [62]. The loss of these anti-inflammatory bacteria may promote a decrease in SCFA production and, consequently, activation of inflammatory pathways. Regarding these metabolites, special mention must be given to acetate, propionate, and butyrate—the latter acting as an energy source for the colon enterocytes, and also intervening in regulation of the intercellular junctions that maintain the intestinal barrier [63]. These molecules possess anti-inflammatory capacity, inhibiting the NF-kB pathway, and they have been shown to reduce arterial pressure [64] and participate in the inhibition of mechanisms implicated in the generation of left ventricular hypertrophy and myocardial fibrosis [65]. Modrego et al. (2023) [34] demonstrated a positive correlation between SCFA levels and LVEF and with anti-inflammatory markers, as well as a negative correlation between butyrate and acetate levels and N-terminal pro b-type natriuretic peptide.

### 4.3. Metagenomic Functional Insights in Heart Failure

In the metagenomic study by Cui, an increase in bacteria carrying genes encoding choline TMA-lyase, choline TMA-lyase-activating enzyme, betaine reductase, or tryptophanase was detected, as well as a decrease in bacteria carrying genes encoding butyrate-acetoacetate CoA transferase, propionate CoA transferase, or formate-tetrahydrofolate ligase. Furthermore, the researchers detected changes in metabolites in patients with HF through metagenomics. They found an increase in two metabolites, including para-Tolyl octanoate, which was significantly elevated in chronic HF patients, while 206 other metabolites, such as niacin, cinnamic acid, and orotic acid, were significantly decreased in chronic HF compared with controls. They also correlated metabolite findings with the genera identified in HF patients versus controls. For example, genera such as Veillonella were negatively correlated with metabolites like niacin, cinnamic acid, and orotic acid, but positively correlated with genera such as Faecalibacterium, Butyricicoccus, and Oscillibacter. The highest proportion of the plasma metabolite sphingosine 1-phosphate was positively correlated with *Veillonella*, *Coprobacillus*, and *Streptococcus*. These findings begin to shed light on the pathophysiology of the gut microbiota in patients with HF, showing a positive correlation between proinflammatory bacteria and metabolites, as well as an inverse correlation between anti-inflammatory bacteria and proinflammatory metabolites [21].

Thanks to the metagenomic data and the finding that half of the studies report an increased relative abundance of *Streptococcus* in patients with HF, our research group suggests, for the first time, how this genus may modulate the pathophysiology of HF. The increase in *Streptococcus* is associated with a higher presence of peptidoglycan and lipoteichoic acid, which may activate inflammatory pathways and, consequently, contribute to an elevated pathophysiological risk of developing HF. Plasma levels of the metabolite sphingosine 1-phosphate may be implicated in the pathophysiology. This genus emerges as an important target to consider in the heart failure population due to its potentially detrimental role.

### 4.4. Dietary Interventions for the Intestinal Microbiota in Heart Failure

Research has started on modulation of the intestinal microbiota in patients with HF. With regard to the diet, meat restriction has been shown to reduce TMAO precursors [66], while the consumption of fruits, vegetables, and whole grains, rich in fiber and polyphenols, contributes to the integrity of the intestinal barrier, reducing LPS and promoting SCFA synthesis [67]. Low-sodium diets have been associated with the improvement of neurohormonal and inflammatory biomarkers [68], while diets containing abundant sodium [69] or fats [70] have been related to dysbiosis. Standards such as the Mediterranean diet or vegetarian or vegan diets have been associated with lower concentrations of TMAO compared with other types of diets [71,72,73,74]. In the case of probiotic agents, nonclinical evidence suggests benefits for heart structure and function [75,76], although a meta-analysis of human studies has not evidenced such effects [77]. Other approaches include physical exercise, which appears to increase microbial diversity [78,79]; fecal transplantation, with the potential to modulate metabolites such as TMAO [80]; and antibiotics such as rifaximin, which was not found to offer benefits in a clinical trial [76].

This review includes 14 studies, of which 9 (65%) were conducted in China and Japan. The populations from different geographic regions may exhibit variations in gut microbiota composition due to underlying cultural diversity. Thus, differences in diet, antibiotic consumption, or physical activity may play a fundamental role when analyzing the gut microbiota. Further studies assessing dietary patterns are needed, as only five of the included studies provided information regarding diet type.

In sum, patients with HF present changes in the composition of their intestinal microbiota at the expense of an increase in bacteria that play a key role in the production of metabolites that exert harmful effects through systemic inflammation. The genus *Streptococcus* increases systemic inflammation due to the presence of peptidoglycan and lipoteichoic acid in its structure. The genera *Escherichia-Shigella* and *Klebsiella* in turn contribute to inflammation by increasing the circulating levels of LPS, TMAO, and IS. On the other hand, patients with HF show a decrease in bacteria that protect against inflammation, such as *Faecalibacterium*, *Blautia*, and *Lachnospira*. The decreased presence of these bacteria results in greater systemic inflammation due to the lower plasma SCFA levels.

We have read with great interest the article by Simadibrata et al. While both studies share a similar focus, our review provides several novel contributions. Specifically, we included five additional studies published in 2023 from diverse regions (China, Japan, Australia, and Spain), increasing the pooled sample size to 550 HF patients and 617 controls. Our review uniquely evaluates microbial evenness, integrates metabolite-related mechanisms (TMAO, indoxyl sulfate, and lipoteichoic acid), and offers the first quantitative synthesis of taxonomic changes across all hierarchical levels (phylum to species). Moreover, our review is the first to hypothesize that the genus *Streptococcus* may have particular relevance in the pathophysiology of HF, as it is increased in half of the included studies, and its association with lipoteichoic acid production could play a key role in systemic inflammation in HF. These additions enhance the comprehensiveness and interpretative value of our work compared to previous reviews [81].

## 5. Limitations

The main limitation of the present systematic review is the heterogeneity of the methods used by the different studies analyzed. Only one study (Cui et al.) employed metagenomic sequencing, while the rest of the studies in our review used 16S rRNA sequencing. Furthermore, there was variability in the regions of the rRNA gene analyzed: three studies focused on the V1-V2 region, six studies on the V3-V4 region, and three studies on the V4-V5 region. This heterogeneity in sequencing methods and rRNA regions analyzed may limit the depth of the taxonomic classification and contribute to discrepancies between studies. This means that no adequate inferential statistical analysis can be made. The heterogeneous description of the different taxa of the intestinal microbiota may limit the conclusions drawn. Another limitation to be taken into account refers to the geographical distribution and dietary and cultural habits of the different populations analyzed, and which could modify the profile of the intestinal microbiota of the individuals studied.

## 6. Conclusions

The current evidence points to the existence of dysbiosis in patients with HF. The microbiota of these individuals is characterized by lower alpha and beta diversity, with an increased presence of bacteria that promote inflammation, and a decrease in other bacteria that exert an anti-inflammatory effect. This modification of the intestinal flora contributes to systemic inflammation and a poorer prognosis of the disease. The findings from the observational studies analyzed suggest that the gut microbiota may play a role in the pathophysiology of HF. In the absence of experimental validation targeting gut microbiota modulation, future studies are needed to evaluate whether exogenous modulation of the gut microbiota could provide clinical benefit in HF.

## Figures and Tables

**Table 1:** Characteristics of patients with heart failure studied in each of the articles that analyzed their intestinal microbiota.

Author	Year	Country	Journal	Case/Control	n	Male, n (%)	Age, Years	BMI, kg/m^2^	HT, n (%)	T2DM, n (%)	eGFR, mL/min/1.73m^2^	CRP, mg/L	LVEF, %	BNP, ng/L	NTproBNP, ng/L	Sequencing Method	NOS-xs
Kamo et al. [27]	2017	Japan	*PLoS One*	HF < 60 y (HFpEF 0%)	12	11 (92)	47.4 ± 2.8	22.9 ± 1.2	1 (8)	4 (33)	54.7 ± 3.1	NR	20.0 ± 2.2 **	1060.6 ± 238.8	NR	16S rRNA (V1-V2)	6
				HF > 60 y (HFpEF 40%)	10	7 (70)	73.8 ± 2.8 *	24.9 ± 1.7	6 (60)	3 (30)	40.4 ± 7.4	NR	43.1 ± 5.8	697.7 ± 176.0	NR		
				HC	12	9 (75)	41.4 ± 2.0	23.2 ± 0.6	0 (0)	0 (0)	NR	NR	NR	NR	NR		
Luedde et al. [32]	2017	Germany	*ESC Heart Fail*	HFrEF	20	11 (55)	65 ± 3.2	29.7 ± 1.4	14 (70)	7 (35)	NR	11.1 ± 2.1 *	22.3 ± 2.9	NR	6564,5 ± 1187,23 *	16S rRNA (V1-V2)	7
				HC	20	11 (55)	65 ± 3.1	29.1 ± 1.3	8 (40)	3 (15)	NR	4.2 ± 1.1	NR	NR	109,2 ± 45,91		
Cui et al. [21]	2018	China	*Sci Rep*	HFrEF	53	44 (83)	58.1 ± 13.3	24.4 ± 4.5	30 (57) *	15 (28) *	NR	3.4 (2.3–8.4) *	29.8 ± 6.6	NR	NR	Metagenomic sequencing	7
				HC	41	32 (78)	53.7 ± 5.9	25.2 ± 3.3	0 (0)	2 (5)	NR	2 (1–3)	NR	NR	NR		
Katsimichas et al. [28]	2018	Japan	*Circ J*	HFrEF	28	21 (75)	51 ± 10 *	21.7 ± 3.4	NR	NR	64.4 ± 25.2 *	6.7 (3.8–35.2) *	25 ± 9 *	375 (145–630) *	NR	16S rRNA (V1-V2)	8
				HC	19	15 (84.2)	36 ± 6	21.8 ± 1.9	NR	NR	87.0 ± 16.9 *	2.9 (1.9–3.8)	62 ± 4	2 (2–6)	NR		
Hayashi et al. [20]	2019	Japan	*Circ J*	Decomp HF	22	14 (64)	72 ± 18	25.8 ± 7.1 †	21 (95)	8 (36)	48.8 ± 19.4	NR	42 ± 17 *	445 (328–763) *,†	NR	16S rRNA (V3-V4)	7
				Comp HF				23.6 ± 5.9			49.0 ± 22.5	NR		284 (152–378) *	NR		
				HC	11	6 (55)	72 ± 7	24.4 ± 3.1	9 (82)	5 (45)	54.0 ± 11.7	NR	63 ± 4	53 (23–109)	NR		
Mayerhofer et al. [33]	2020	Norway	*ESC Heart Fail*	HFrEF	84	34 (40.5)	59 (39–74) *	27.9 (26.8–29.1)	25 (29.8) *	18 (21.4) *	68.9 (64.3–73.5)	3.3 (2.3–4.4)	28.2 ± 7.3	NR	2664.1 (1726.2–3602.0)	16S rRNA (V3-V4)	7
				HC	266	107 (40.2)	46 (30–61)	26.4 (25.9–26.9)	11 (4.1)	2 (0.8)	NR	NR	NR	NR	NR		
Beale et al. [30]	2021	Australia	*J Am Heart Assoc*	HFpEF	26	6 (23) ‡	68 ± 7.5 *	32.8 ± 5.8 *	18 (69) *	4 (15) *	NR	NR	60.5 ± 5.1	NR	NR	16S rRNA (V4-V5)	8
				Metropolitan HC	39	22 (56)	58.3 ± 7.9	25.1 ± 2.9	15 (38)	0 (0)	NR	NR	NR	NR	NR		
				Regional HC	28	9 (32)	61 ± 6	25.3 ± 2.5	5 (18)	0 (0)	NR	NR	NR	NR	NR		
Wang et al. [22]	2021	China	*Mediators Inflamm*	HF	25	14 (56)	65 ± 3.2	29.7 ± 1.4	NR	NR	NR	11.1 ± 2.1 *	NR	NR	6564.5 ± 1187.2 *	16S rDNA (V3-V4)	5
				HC	25	13 (52)	65 ± 3.1	29.1 ± 1.3	NR	NR	NR	4.2 ± 1.1	NR	NR	109.2 ± 45.9		
Huang et al. [23]	2022	China	*Front Cardiovasc Med*	HFpEF	30	19 (63.3)	71.2 ± 9.4	23.8 ± 3	25 (83.3)	NR	NR	NR	NR	NR	NR	16S rRNA (V4-V5)	5
				HC	30	17 (56.7)	67 ± 7.4	23.9 ± 3	NR	NR	NR	Nr	NR	NR	NR		
Sun et al. [24]	2022	China	*Front Microbiol*	HF	29	24 (82.8) *	60.7 ± 11.7	24.0 ± 3.5	14 (48)	10 (34)	NR	NR	33.8 ± 9.1*	NR	4745.7 (1130–16,755) *	16S rRNA (V3-V4)	5
				HC	30	10 (33.3)	60 ± 9.6	24.9 ± 3.1	11 (37)	5 (16.7)	NR	NR	63.2 ± 4.7	NR	124 (25–258)		
Zhang et al. [25]	2023	China	*Front Cardiovasc Med*	HF NYHA III	29	26 (44.8) *	77 (73.5–83.5)	NR	19 (65.5)	NR	NR	NR	63 (42.5–66.5) *	1014.7 (802.6–1321) *	NR	16S rRNA (V3-V4)	5
				HF NYHA IV	29		79 (69.5–86.5)	NR	17 (58.6)	NR	NR	NR	43 (38.5–50) *	2789.2 (2256.8–3781.6) *	NR		
				HC	22	13 (59)	76 (73.8–80)	NR	13 (59)	NR	NR	NR	67 (62–71.3)	42.5 (15.5–102.7)	NR		
Peng et al. [26]	2023	China	*Front Cell* *Infect Microbiol*	No sarcopenia HF	33	24 (72.7)	71.8 ± 7.9	24.2 ± 2.8	NR	NR	NR	NR	57 (39.5–61.5) *	NR	1084 (372–3200) *	16S rRNA (V3-V4)	5
				Sarcopenia HF	29	13 (44.8)	75.1 ± 8.2	20.7 ± 3.8 *	NR	NR	NR	NR	55 (38–60) *	NR	1424 (514–4830) *		
				HC	15	8 (53.3)	67.7 ± 9.8	23.5 ± 3.1	NR	NR	NR	NR	63 (60–65)	NR	73.6 (27.1–120)		
Ahmad et al. [31]	2023	Australia	*Am J Physiol Heart* *Circ Physiol*	HFrEF	73	61 (83.5) *	59.8 ± 12.4	30.71 ± 6.1 *	31 (42.5)	20 (27.4)	NR	35.9 ± 46.3	29.5 ± 9.4	765.2 ± 1051.1	2983.7 ± 5245.5	16S rRNA (V3-V4)	8
				HC	59	11 (18.6)	56.0 ± 9.2	26.4 ± 3.9	NR	NR	NR	NR	NR	NR	NR		
Modrego et al. [34]	2023	Spain	*Int J Mol Sci*	de novo HF	18	7 (38.9)	67.6 ± 4.1	NR	NR	7 (38.9)	NR	7.5 (3.7–22.2) §	36.2 ± 4.3 §	NR	7081 ± 3544 §	16S rRNA	7
				12-months follow-up			68.3 ± 4.3	NR	NR		NR	2.9 (2.7–5.3)	56.7 ± 3.5	NR	358 ± 69		

BMI: body mass index; BNP: brain natriuretic peptide; CRP: C-reactive protein; eGFR: estimated glomerular filtration rate; HC: healthy controls; HFpEF: heart failure with preserved ejection fraction; HFrEF: heart failure with reduced ejection fraction; HT: hypertension; LVEF: left ventricular ejection fraction; NOS-xs: cross-sectional Newcastle-Ottawa scale; NR: Not Reported; NTproBNP: N-terminal prohormone of brain natriuretic peptide; T2DM: type 2 diabetes mellitus. *: *p* < 0.05 HF patients vs. HC; **: *p* < 0.05 HF patients under 60 years vs. HF patients over 60 years; †: decomp HF patients vs. comp HF patients; ‡: *p* < 0.05 HF patients vs. metropolitan HC group; §: *p* < 0.05 HF patients at beginning vs. HF after 12-months follow-up. The values express n (%) for dichotomic variables, the median (interquartile range) for continuous variables with a non-normal distribution, and the mean ± standard deviation for continuous variables with a normal distribution.

**Table 2:** Differences in alpha and beta diversity between the patients with heart failure and the controls, and according to left ventricular ejection fraction.

	Alpha Diversity			Beta Diversity
	Overall Diversity	Richness	Evenness	
HFpEF + HFrEF				
Kamo et al. (2017) [27]	(=)	(=)	NR	Significant differences between groups
Hayashi et al. (2019) [29]	(=)	NR	NR	NR
Wang et al. (2021) [22]	↓	↓	↓	Significant differences between groups
Sun et al. (2022) [24]	↓	↓	↓	Significant differences between groups
Zhang et al. (2023) [25]	↓	↓	NR	Significant differences between groups
Peng et al. (2023) [26]	↓	↓	↓	Significant differences between groups
Modrego et al. (2023) [34]	(=)	NR	(=)	No differences between baseline vs. 12-months follow-up
HFpEF				
Beale et al. (2021) [30]	↓	↓	NR	Significant differences between groups
Huang et al. (2022) [23]	↓	↓	(=)	Significant differences between groups
HFrEF				
Luedde et al. (2017) [32]	↓	(=)	NR	Significant differences between groups
Cui et al. (2018) [21]	NR	NR	NR	Significant differences between groups
Katsimichas et al. (2018) [28]	(=)	(=)	(=)	Significant differences between groups
Mayerhofer et al. (2020) [33]	NR	↓	NR	Significant differences between groups
Ahmad et al. (2023) [31]	↓	NR	NR	NR

(=) similar; ↓ decreased; HFpEF: heart failure with preserved ejection fraction; HFrEF: heart failure with reduced ejection fraction; NR: not reported.

**Table 3:** Differences observed in each taxonomic group between patients with heart failure and healthy controls.

Phylum	Class	Family	Genus	Species
Bacillota (Firmicutes) = [27], ↓ [24,25,26,33]	Clostridia ↓ [26,31]	Clostridiaceae	*Clostridium* ↓ [27]	
			SMB53 ↓ [28]	
		Ruminococcaceae ↑ [22], ↓ [24,30,31]	*Ruminococcus* = [27], ↑ [21,22], ↓ [30]	*Ruminococcus gnavus* ↑ [21]
			*Faecalibacterium* = [27], ↓ [21,24,26,32,33]	*Faecalibacterium prausnitzii* ↓ [21]
			*Oscillibacter* ↓ [21]	*Oscillibacter* sp. ↓ [21]
			*Butyricicoccus* ↓ [23]	
			*Ruminiclostridium* ↓ [23]	
			*Uncl. Ruminococcaceae* ↓ [32]	
		Lachnospiraceae ↓ [24,25,31,33]	*Lachnospira* ↓ [23,31]	
			*Blautia* = [27], ↓ [26,31,33]	
			*Anaerostipes* = [27], ↓ [33]	
			*Dorea* ↓ [27]	
			*Hungatella* ↑ [33]	
			*Fusicatenibacter* ↓ [33]	
			*Pseudobutyrivibrio* ↓ [33]	
			*Coprococcus* ↓ [33]	
			*Agathobacter* ↓ [25]	
		Caldicellulosiruptoraceae	*Caldicellulosiruptor* ↓ [30]	
		Eubacteriaceae	*Eubacterium* ↓ [27,33]	*Eubacterium rectale* ↓ [27]
		Peptostreptococcaceae ↓ [26]	*Romboutsia* ↑ [22]	
	Negativicutes	Veillonellaceae	*Veillonella* ↑ [21,28]	*Veillonella* sp. ↑ [21]
			*Megamonas* ↑ [31] **, ↓ [25,29]	
			*Megasphaera* ↑ [30]	
			*Mitsuokella* ↓ [30]	
			*Dialister* ↓ [24]	
		Acidaminococcaceae	*Acidaminococcus* ↑ [34] *	
			*Succiniclasticum* ↑ [33]	
	Erysipelotrichia	Erysipelotrichaceae ↓ [32]	*Uncl. Erysipelotrichaceae* ↓ [32]	
			L7A_E11 ↓ [30]	
	Bacilli	Enterococcaceae ↑ [24]	*Enterococcus* ↑ [23,24]	
		Leuconostocaceae ↑ [26]		
		Streptococcaceae	*Streptococcus* ↑ [21,22,25,27,28,30]	*Streptococcus* sp. ↑ [21]
		Lactobacillaceae	*Lactobacillus* ↑ [21,22,23,25,27]	
			*Lacticaseibacillus* ↑ [31] §	
		Ammoniphilaceae	*Ammoniphilus* ↑ [30]	
		RF39 ↓ [34] *		
	Mollicutes	Acholeplasmataceae	*Acholeplasma* ↑ [30]	
Bacteroidota (Bacteroidetes) = [25,27], ↑ [33], ↓ [24,26]	Bacteroidia	Prevotellaceae ↓ [26]	*Prevotella* = [27], ↑ [31] **, [33], ↓ [26]	
		Barnesiellaceae ↑ [31] **		
		Bacteroidaceae	*Bacteroides* = [27], ↑ [30]	
		Tannerellaceae	*Parabacteroides* = [27]	
		Rikenellaceae	*Alistipes* ↓ [21]	
Actinomycetota (Actinobacteria) = [27], ↑ [24,26,29]	Coriobacteriia	Coriobacteriaceae ↓ [32]	*Collinsella* = [27], ↓ [32]	
		Atopobiaceae	*Atopobium* ↑ [22]	
			*Libanicoccus* ↑ [31] **	
		Eggerthellaceae	*Slackia* ↑ [26]	
	Actinomycetes	Nocardiaceae ↑ [26]		
		Pseudonocardiaceae ↑ [26]		
		Bifidobacteriaceae	*Bifidobacterium* = [27], ↑ [25], [29] †, [34] *, ↓ [33]	
Pseudomonadota (Proteobacteria) = [27], ↑ [24,26], ↓ [34] *	Alphaproteobacteria ↑ [26]	Sphingosinicellaceae	*Sphingosinicella* ↓ [34] *	
		Sphingomonadaceae	*Sphingomonas* ↓ [34] *	
		Bradyrhizobiaceae	*Bradyrhizobium* ↓ [34] *	
	Betaproteobacteria	Sutterellaceae	*Sutterella* ↑ [30], ↓ [23]	*Sutterella wadsworthensis* ↓ [21]
	Gammaproteobacteria	Enterobacteriaceae ↑ [25]	*Escherichia-Shigella* = [27], ↑ [22,24,25], [29] ‡, [32]	
			*Klebsiella* = [27], ↑ [22,24,25]	
		Pasteurellaceae	*Haemophilus* ↑ [22]	
		Erwiniaceae	*Erwinia* ↑ [30]	
		Moraxellaceae	*Acinetobacter* ↑ [21]	
		Pectobacteriaceae	*Pectobacterium* ↓ [34] *	
Synergistota ↑ [27]				
Verrucomicrobiota	Verrucomicrobiae	Akkermansiaceae	*Akkermansia* ↑ [30]	

* 12 months follow-up vs. admission; ** Chronic HF vs. Acute HF 6 months follow-up; † Comp-HF vs. HC; ‡ Decomp-HF vs. Comp-HF; § Chronic HF vs. Acute HF 6 months baseline.

**Table 4:** Summary of the main findings on the proportion of studies reporting significant taxonomic reclassifications among predominant bacterial genera.

Phylum		Class		Family		Genus	
**Bacillota**	**↓** **29%**	**Clostridia**	**↓** **14%**	**Lachnospiraceae**	**↓** **30%**	**Lachnospira**	**↓** **15%**
						**Blautia**	**↓** **23%**
				**Ruminococcaceae**	**↓** **21%**		
				Eubacteriaceae		**Eubacterium**	**↓** **15%**
				Oscillospiraceae		**Faecalibacterium**	**↓** **38%**
		Bacilli		Streptococcaceae		**Streptococcus**	**↑** **46%**
				Lactobacillaceae		**Lactobacillus**	**↑** **38%**
				Enterococcaceae		**Enterococcus**	**↑** **15%**
**Pseudomonadota**	**↑** **14%**	Gammaproteobacteria		Enterobacteriaceae		**Escherichia-Shigella**	**↑** **38%**
						**Klebsiella**	**↑** **23%**
		**Alphaproteobacteria**	**↑** **7%**				
**Actinomycetota**	**↑** **21%**						
**Synergistota**	**↑** **7%**						

Values in bold indicate the proportion of studies that reported taxonomic changes at different hierarchical levels. ↓: Proportion of studies showing a decrease at each specified taxonomic rank; ↑: Proportion of studies showing a increase at each specified taxonomic rank.

**Table 5:** Key determinants of gut microbiota modulation identified in the included studies.

Author	Country	Antibiotics	Probiotics	Proton Pump Inhibitors	Diuretics	Diet
Kamo et al., 2017 [27]	Japan	Excluded if taken 2 months before	Excluded if taken 2 months before	Collected No analysis	Not collected	Not collected
Luedde et al., 2017 [32]	Germany	Excluded if taken 3 months before	Excluded if taken 3 months before	Not collected	Collected Multivariate analysis	All HF patients and HC consumed a mixed European diet
Cui et al., 2018 [21]	China	Excluded if taken 1 month before	Excluded if taken 1 month before	Collected Multivariate analysis	Collected as part of the study population characteristics	Not collected
Katsimichas et al., 2018 [28]	Japan	Excluded if taken 1 month before	Excluded if taken 1 month before	Collected Multivariate analysis	Not collected	Analysis of diet differences between HF and HC groups
Hayashi et al., 2019 [20]	Japan	Excluded if taken 1 month before	Not collected	Collected Analysis of differences in genera between users vs. non-users	Collected as part of the study population characteristics	Not collected
Mayerhofer et al., 2020 [33]	Norway	Excluded if taken 3 months before	Not collected	Not collected	Collected as part of the study population characteristics	Collected in HF validation group Correlation analysis between diet, bacteria and metabolites
Beale et al., 2021 [30]	Australia	Excluded if taken 3 months before	Excluded if taken 3 months before	Not collected	Collected as part of the study population characteristics	Multivariable Analysis between HF patients and HC
Wang et al., 2021 [22]	China	Not collected	Not collected	Not collected	Not collected	Not collected
Huang et al., 2022 [23]	China	Excluded if taken 3 months before	Excluded if taken 3 months before	Not collected	Not collected	Not collected
Sun et al., 2022 [24]	China	Excluded if taken 1 month before	Excluded if taken 1 month before	Not collected	Not collected	Not collected
Zhang et al., 2023 [25]	China	Excluded if taken 3 months before	Excluded if taken 3 months before	Not collected	Collected as part of the study population characteristics	Not collected
Peng et al., 2023 [26]	China	Excluded if taken 1 month before	Excluded if taken 1 month before	Not collected	Collected as part of the study population characteristics	Not collected
Ahmad et al., 2023 [31]	Australia	Excluded if taken 3 months before	Excluded if taken 3 months before	Not collected	Collected as part of the study population characteristics	Not collected
Modrego et al., 2023 [34]	Spain	Excluded if taken 2 months before	Excluded if taken 2 months before	Not collected	Collected as part of the study population characteristics	Analysis of diet differences between admission and 12 months follow-up

HC: healthy controls; HF: heart failure.

**Table 6:** Main changes in metabolites derived from gut microbiota alterations in patients with heart failure compared with controls.

Increase	Decrease
Para-Tolyl octanoate [21]	Niacin [21]
Homocitrulline [22]	Cinnamic acid [21]
Sphingosine 1− phosphate [21]	Orotic acid [21]
Ethylsalicylate [22]	Riboflavin [22]
Acetate [31]	Citramalate [22]
Phenylacetylglutamine [25]	Biocytin [22]
Soluble CD14 [31]	Indoxyl sulfate [29]
Trimethylamine N-oxide [29,31]	

## Supplementary Materials

The following supporting information can be downloaded at: https://www.mdpi.com/article/10.3390/jcm14228110/s1, Supplementary Material S1: PRISMA 2020 checklist.

## Abbreviations

HR	Hazard ratio
HF	Heart failure
IS	Indoxyl sulfate
IL-6	Interleukin 6
LVEF	Left ventricular ejection fraction
LPS	Lipopolysaccharide
NF-kB	Nuclear factor kappa-light-chain-enhancer of activated B cells
RoB	Risk of bias
SCFA	Short-chain fatty acids
NHE3	Sodium-Hydrogen exchanger 3
TLRs	Toll-like receptors
TGF-β1	Transforming growth factor beta 1
TMA	Trimethylamine
TMAO	Trimethylamine N-oxide
TNF-α	Tumor necrosis factor alpha
