# Supplementary material for: Gut Microbiota Alterations in Heart Failure Patients: Insights from a Systematic Review
Source: J Clin Med. 2025 Nov 16;14(22):8110. doi: 10.3390/jcm14228110 (PMC12653670; doi:10.3390/jcm14228110)
Supplement: Supplementary file 1 [file jcm-14-08110-s001.zip › jcm-3937704-supplementary.pdf]

# PRISMA 2020 Checklist

| Section and Topic | Item # | Checklist item | Location where item is reported |
|---|---|---|---|
| **TITLE** | | | |
| Title | 1 | Identify the report as a systematic review. | Inclusion criteria: observational studies or clinical trials. Exclusion criteria: letters to the editor, editorials, short communications, animal studies, narrative reviews, systematic reviews, meta-analyses and studies involving interventions to modulate the microbiota. |
| **ABSTRACT** | | | |
| Abstract | 2 | See the PRISMA 2020 for Abstracts checklist. | |
| **INTRODUCTION** | | | |
| Rationale | 3 | Describe the rationale for the review in the context of existing knowledge. | There is limited scientific evidence on gut dysbiosis in patients with heart failure. Therefore, we propose a review of the available literature to summarize the current findings. |
| Objectives | 4 | Provide an explicit statement of the objective(s) or question(s) the review addresses. | We decided to evaluate the |

# PRISMA 2020 Checklist

| Section and Topic | Item # | Checklist item | Location where item is reported |
|---|---|---|---|
| | | | different taxonomic levels of the gut microbiota across the various studies on heart failure. |
| **METHODS** | | | |
| Eligibility criteria | 5 | Specify the inclusion and exclusion criteria for the review and how studies were grouped for the syntheses. | We included included all original studies of the intestinal microbiota using gene sequencing methods in patients with HF, published in English between July 2015 and July 2025 |
| Information sources | 6 | Specify all databases, registers, websites, organisations, reference lists and other sources searched or consulted to identify studies. Specify the date when each source was last searched or consulted. | PubMed/MEDLINE |
| Search strategy | 7 | Present the full search strategies for all databases, registers and websites, including any filters and limits used. | The search strategy made use of MeSH terms and keywords combined using boolean operators, based on the following equation: ("Heart Failure" OR "heart failure with reduced ejection fraction" OR "heart failure |

| Section and Topic | Item # | Checklist item | Location where item is reported |
|---|---|---|---|
| | | | with preserved ejection fraction") AND ("Gut microbiota" OR "intestinal microbiota" OR "gut dysbiosis" OR "microbiome" OR "gut flora"). |
| Selection process | 8 | Specify the methods used to decide whether a study met the inclusion criteria of the review, including how many reviewers screened each record and each report retrieved, whether they worked independently, and if applicable, details of automation tools used in the process. | Inclusion criteria: observational studies or clinical trials. Exclusion criteria: letters to the editor, editorials, short communications, animal studies, narrative reviews, systematic reviews, meta-analyses and studies involving interventions to modulate the microbiota. |
| Data collection process | 9 | Specify the methods used to collect data from reports, including how many reviewers collected data from each report, whether they worked independently, any processes for obtaining or confirming data from study investigators, and if applicable, details of automation tools used in the process. | The two main authors conducted the search independently, without using artificial intelligence. |

# PRISMA 2020 Checklist

| Section and Topic | Item # | Checklist item | Location where item is reported |
|---|---|---|---|
| | | | Together, they created the flow chart. The risk of bias was minimized using the Newcastle-Ottawa Scale. |
| Data items | 10a | List and define all outcomes for which data were sought. Specify whether all results that were compatible with each outcome domain in each study were sought (e.g. for all measures, time points, analyses), and if not, the methods used to decide which results to collect. | We retrieved all available taxonomic levels reported in each study. The results were then classified according to their respective taxonomic levels. |
| | 10b | List and define all other variables for which data were sought (e.g. participant and intervention characteristics, funding sources). Describe any assumptions made about any missing or unclear information. | No sources of funding were required for this work |
| Study risk of bias assessment | 11 | Specify the methods used to assess risk of bias in the included studies, including details of the tool(s) used, how many reviewers assessed each study and whether they worked independently, and if applicable, details of automation tools used in the process. | The risk of bias (RoB) of each of the studies was evaluated independently by three of the authors, using the Newcastle-Ottawa scale for cross-sectional studies (NOS-xs)[16]. The total score classified the studies as presenting high |

# PRISMA 2020 Checklist

| Section and Topic | Item # | Checklist item | Location where item is reported |
|---|---|---|---|
| | | | RoB (0-3 points), moderate RoB (4-6 points) or low RoB (7-9 points). |
| Effect measures | 12 | Specify for each outcome the effect measure(s) (e.g. risk ratio, mean difference) used in the synthesis or presentation of results. | Given the descriptive nature of the findings, it was not possible to extract association variables. The findings were described in a cross-sectional manner only. |
| Synthesis methods | 13a | Describe the processes used to decide which studies were eligible for each synthesis (e.g. tabulating the study intervention characteristics and comparing against the planned groups for each synthesis (item #5)). | The characteristics of the included studies were compared with each other in Table 1. |
| | 13b | Describe any methods required to prepare the data for presentation or synthesis, such as handling of missing summary statistics, or data conversions. | The results reported in each of the studies were highly heterogeneous. |
| | 13c | Describe any methods used to tabulate or visually display results of individual studies and syntheses. | Relevant overlapping results from multiple studies were detailed in the Results section of the text. The remaining findings were |

| Section and Topic | Item # | Checklist item | Location where item is reported |
|---|---|---|---|
| | | | described in Table 1. |
| | 13d | Describe any methods used to synthesize results and provide a rationale for the choice(s). If meta-analysis was performed, describe the model(s), method(s) to identify the presence and extent of statistical heterogeneity, and software package(s) used. | We grouped the findings based on the similarities observed between studies across different taxonomic levels. |
| | 13e | Describe any methods used to explore possible causes of heterogeneity among study results (e.g. subgroup analysis, meta-regression). | The methodologies of the different studies are described in the Results section. |
| | 13f | Describe any sensitivity analyses conducted to assess robustness of the synthesized results. | No sensitivity analyses were conducted. |
| Reporting bias assessment | 14 | Describe any methods used to assess risk of bias due to missing results in a synthesis (arising from reporting biases). | Since all published studies were collected, we do not anticipate publication bias or missing results. All available studies meeting the inclusion criteria were included. |
| Certainty assessment | 15 | Describe any methods used to assess certainty (or confidence) in the body of evidence for an outcome. | We analyzed the methodology of the bacterial genomic studies. All but one used the same |

| Section and Topic | Item # | Checklist item | Location where item is reported |
|---|---|---|---|
| | | | methodology, which is appropriate for clinical practice. |
| **RESULTS** | | | |
| Study selection | 16a | Describe the results of the search and selection process, from the number of records identified in the search to the number of studies included in the review, ideally using a flow diagram. | The initial search identified 307 articles. Of these, 202 were excluded after reading the title and abstract. Following full-text reading and applying the exclusion criteria, 6 observational studies that were not focused on the objective of our review were discarded, along with 3 clinical trial protocols, 67 narrative reviews, 5 systematic reviews and 7 meta-analyses. |
| | 16b | Cite studies that might appear to meet the inclusion criteria, but which were excluded, and explain why they were excluded. | we excluded the study published by Pasini et al. (2016)[18], as it did not use gene sequencing; |

| Section and Topic | Item # | Checklist item | Location where item is reported |
|---|---|---|---|
| | | | the article published by Kummen et al. (2018)[19], due to insufficient data on the included subjects; and the article published by Hayashi et al. (2021)[20], which involved the same database as that of a previous study from 2019, with a different objective. |
| Study characteristics | 17 | Cite each included study and present its characteristics. | Kamo et al., 2017, PLoS One. Comparison of patients with heart failure versus controls. Wang et al., 2021, Mediators Inflamm. Comparison of patients with heart failure versus controls. Huang et al., 2022, Front Cardiovasc Med. Comparison of patients with heart failure versus controls. Sun et al., 2022, |

# PRISMA 2020 Checklist

| Section and Topic | Item # | Checklist item | Location where item is reported |
|---|---|---|---|
| | | | Front Microbiol. Comparison of patients with heart failure versus controls. Zhang et al., 2023, Front Cardiovasc Med. Comparison of patients with heart failure with symptoms upon exertion versus patients with heart failure with symptoms at rest, and both compared to healthy controls. Peng et al., 2023, Front Cell Infect Microbiol. Comparison of patients with heart failure and sarcopenia versus patients with heart failure without sarcopenia and versus controls. Mayerhofer et al., 2020, ESC Heart Fail. Comparison of patients with heart failure |

# PRISMA 2020 Checklist

| Section and Topic | Item # | Checklist item | Location where item is reported |
|---|---|---|---|
| | | | versus controls. Beale et al., 2021, J Am Heart Assoc. Comparison of patients with heart failure versus controls. Ahmad et al., 2023, Am J Physiol Heart Circ Physiol. Comparison of patients with heart failure versus controls. Modrego et al., 2023, Int J Mol Sci. Comparison of patients with heart failure during a hospital admission versus patients with heart failure after 12 months. Luedde et al., 2017, ESC Heart Fail. Comparison of patients with heart failure versus controls. Cui et al., 2018, Sci Rep. Comparison of patients with heart failure versus controls. |

| Section and Topic | Item # | Checklist item | Location where item is reported |
|---|---|---|---|
| | | | Katsimichas et al., 2018, Circ J. Comparison of patients with heart failure versus controls. Hayashi et al., 2019, Circ J. Comparison of compensated |
| Risk of bias in studies | 18 | Present assessments of risk of bias for each included study. | Six studies were classified as presenting moderate RoB (Kamo et al., 2017, PLoS One; Wang et al., 2021, Mediators Inflamm; Huang et al., 2022, Front Cardiovasc Med; Sun et al., 2022, Front Microbiol; Zhang et al., 2023, Front Cardiovasc Med; Peng et al., 2023, Front Cell Infect Microbiol) and 8 as presenting low RoB (Mayerhofer et al., 2020, ESC Heart Fail; Beale et al., 2021, J Am Heart Assoc; Ahmad et |

| Section and Topic | Item # | Checklist item | Location where item is reported |
|---|---|---|---|
| | | | al., 2023, Am J Physiol Heart Circ Physiol; Modrego et al., 2023, Int J Mol Sci; Luedde et al., 2017, ESC Heart Fail; Cui et al., 2018, Sci Rep; Katsimichas et al., 2018, Circ J; Hayashi et al., 2019, Circ J). |
| Results of individual studies | 19 | For all outcomes, present, for each study: (a) summary statistics for each group (where appropriate) and (b) an effect estimate and its precision (e.g. confidence/credible interval), ideally using structured tables or plots. | Differences in beta diversity were found in all studies except those by Ahmad et al. and Hayashi et al. |
| Results of syntheses | 20a | For each synthesis, briefly summarise the characteristics and risk of bias among contributing studies. | For each synthesis, we described the main characteristics of the included studies, such as study design and sample size. We also assessed the risk of bias in each study using the Newcastle-Ottawa scale, finding that most studies had |

| Section and Topic | Item # | Checklist item | Location where item is reported |
|---|---|---|---|
| | | | low to moderate risk of bias. |
| | 20b | Present results of all statistical syntheses conducted. If meta-analysis was done, present for each the summary estimate and its precision (e.g. confidence/credible interval) and measures of statistical heterogeneity. If comparing groups, describe the direction of the effect. | No statistical analysis or statistical inference was performed on the results obtained. |
| | 20c | Present results of all investigations of possible causes of heterogeneity among study results. | No statistical analysis of heterogeneity was conducted. |
| | 20d | Present results of all sensitivity analyses conducted to assess the robustness of the synthesized results. | No sensitivity analyses were conducted. |
| Reporting biases | 21 | Present assessments of risk of bias due to missing results (arising from reporting biases) for each synthesis assessed. | Given that all published studies meeting the inclusion criteria were included, there is no evidence of reporting bias or missing results. Therefore, the risk of bias due to missing results in the syntheses is considered low. |
| Certainty of evidence | 22 | Present assessments of certainty (or confidence) in the body of evidence for each outcome assessed. | The certainty of the evidence for each outcome was assessed based on the study methodologies |

# PRISMA 2020 Checklist

| Section and Topic | Item # | Checklist item | Location where item is reported |
|---|---|---|---|
| | | | and consistency of findings. Most studies used appropriate genomic sequencing methods and showed consistent results, which supports moderate confidence in the overall evidence. |
| **DISCUSSION** | | | |
| Discussion | 23a | Provide a general interpretation of the results in the context of other evidence. | The main differences in intestinal microbiota between patients with HF and the healthy controls were referred to the phyla Bacillota and Pseudomonadota. Within the phylum Bacillota, a decrease was observed in most of the genera of class Clostridia, particularly Faecalibacterium, as well as an increase in class Bacilli, particularly |

# PRISMA 2020 Checklist

| Section and Topic | Item # | Checklist item | Location where item is reported |
| --- | --- | --- | --- |
| | | | of the genera Streptococcus and Lactobacillus. |
| | 23b | Discuss any limitations of the evidence included in the review. | The main limitation of the present systematic review is the heterogeneity of the methods used by the different studies analyzed. This means that no adequate inferential statistical analysis can be made. The heterogeneous description of the different taxa of the intestinal microbiota may limit the conclusions drawn. Another limitation to be taken into account refers to the geographical distribution and dietary and cultural habits of the different populations analyzed, and which could |

# PRISMA 2020 Checklist

| Section and Topic | Item # | Checklist item | Location where item is reported |
|---|---|---|---|
| | | | modify the profile of the intestinal microbiota of the individuals studied. |
| | 23c | Discuss any limitations of the review processes used. | The review process had some limitations. First, only studies published in certain databases were included, which may have led to selection bias.Lastly, the absence of meta-analysis due to heterogeneity limited the ability to quantitatively synthesize results. |
| | 23d | Discuss implications of the results for practice, policy, and future research. | The findings of this review highlight the potential role of gut microbiota alterations in heart failure, suggesting that microbiome-targeted therapies could be explored in clinical practice. From a policy perspective, |

| Section and Topic | Item # | Checklist item | Location where item is reported |
|---|---|---|---|
| | | | incorporating microbiome research into cardiovascular health guidelines may improve patient management. Future research should focus on longitudinal studies and intervention trials to better understand causality and therapeutic potential. |
| **OTHER INFORMATION** | | | |
| Registration and protocol | 24a | Provide registration information for the review, including register name and registration number, or state that the review was not registered. | This review was not registered. |
| | 24b | Indicate where the review protocol can be accessed, or state that a protocol was not prepared. | No protocol was prepared for this review. |
| | 24c | Describe and explain any amendments to information provided at registration or in the protocol. | No amendments were made to the information initially provided in the registration or protocol, as no registration or protocol was prepared for this review. |
| Support | 25 | Describe sources of financial or non-financial support for the review, and the role of the funders or sponsors in the review. | No funding or financial support was |

| Section and Topic | Item # | Checklist item | Location where item is reported |
|---|---|---|---|
| | | | received for this review. The authors declare no involvement of any funders or sponsors in the design, conduct, or reporting of this study. |
| Competing interests | 26 | Declare any competing interests of review authors. | The authors declare no competing interests. |
| Availability of data, code and other materials | 27 | Report which of the following are publicly available and where they can be found: template data collection forms; data extracted from included studies; data used for all analyses; analytic code; any other materials used in the review. | No publicly available materials such as data collection forms, extracted data, analytic code, or other review materials were generated or used in this review. Only data from the published studies were used. |

*From:* Page MJ, McKenzie JE, Bossuyt PM, Boutron I, Hoffmann TC, Mulrow CD, et al. The PRISMA 2020 statement: an updated guideline for reporting systematic reviews. BMJ 2021;372:n71. doi: 10.1136/bmj.n71.
This work is licensed under CC BY 4.0. To view a copy of this license, visit https://creativecommons.org/licenses/by/4.0/
